# 

**Published:** 1904-07

**Authors:** 


### BOOKS RECEIVED.

Medical Diagnosis. Special Diagnosis of Internal Medicine. A Handbook for Physicians and Students. By Dr. Wilhelm v. Leube, Professor of Medicine, and Physician-in-Chief to the Julius Hospital at Wurzburg. Authorised Translation from the Sixth German Edition. Edited, with Annotations, by Julius L. Salinger, M.D., late Assistant Professor of Clinical Medicine in the Jefferson Medical College, and Physician to the Philadelphia Hospital. Octavo, pp. 1084. With five colored plates and 74 illustrations in the text. New York and London: D. Appleton & Co. 1904. (Price, $5.00.)

American Edition of Nothnagel's Practice. Tuberculosis and Acute General Miliary Tuberculosis. By Dr. G. Cornet, of Berlin. Edited, with additions, by Walter B. James, M.D., Professor of the Practice of Medicine in the College of Physicians and Surgeons (Columbia University), New York. Octavo, 806 pages. Philadelphia, New York, London: W. B. Saunders & Company. 1904. (Cloth, $5.00 net; half morocco, $6.00 net.)

American Edition of Nothnagel's Practice. Diseases of the Intestines and peritoneum. By Dr. Hermann Nothnagel, of Vienna. Edited, with additions, by Humphrey D. Rolleston, M.D., F.R.C.P., Physician to Saint George's Hospital, London, England. Octavo, 1032 pages, fully illustrated. Philadelphia, New York, London: W. B. Saunders & Company. 1904. (Cloth, $5.00 net; half morocco, $6.00 net.)

Modern Ophthalmology. A Practical Treatise on the Anatomy, Physiology, and Diseases of the Eye. By James Moores Ball, M.D., Professor of Ophthalmology in the Saint Louis College of Physicians and Surgeons. Royal octavo, pp. 820. With 417 illustrations and 21 colored plates, nearly all original. Philadelphia: F. A. Davis Company. 1904. (Price: cloth, $7.00 net; half morocco, $8.50 net.)

Annual Report of the Surgeon-General of the Public Health and Marine Hospital Service of the United States for the fiscal year 1903. Washington: Government Printing Office. 1904.

Obstetric and Gynecologic Nursing. By Edward P. Davis, A.M., M.D., Professor of Obstetrics in the Jefferson Medical College and in the Philadelphia Polyclinic. Duodecimo, 402 pages, fully illustrated. Second edition, thoroughly revised. Philadelphia, New York, London: W. B. Saunders & Company. 1904. (Polished buckram, $1.75 net.)

Epilepsy and its Treatment. By William P. Spratling, M.D., Superintendent of the Craig Colony for Epileptics at Sonyea, N. Y. Octavo volume of 522 pages, illustrated. Philadelphia, New York, London: W. B. Saunders & Company. 1904. (Cloth, $4.00 net.)

Clinical Treatises on the Pathology and Therapy of Disorders of Metabolism and Nutrition. By Prof. Dr. Carl von Noorden, Physician-in-Chief to the City Hospital, Frankfurt a. M. Authorised American edition, translated under the direction of Boardman Reed, M.D., Philadelphia. Part V. Concerning the Effects of Saline Waters on Metabolism. New York: E. B. Treat & Company. 1904. (Price, 75 cents.)

Railway and other Accidents with Relation to Injury and Disease of the Nervous System. A Book for Court use. By Allan McLane Hamilton, M.D., F.R.S.E., New York. Octavo, pp. 363. Illustrated. New York: William Wood & Company. 1904. (price, $3.50.)

The Practical Medicine Series of Year Books. Ten volumes. Issued under the general editorial charge of Gustavus P. Head, M.D., Professor of Laryngology and Rhinology in the Chicago Post-Graduate Medical School. Volume V. Obstetrics. Edited by Joseph B. DeLee, M.D., Professor of Obstetrics, Northwestern University Medical School. Chicago: The Year Book Publishers. 1904. (Price, $1.00; entire series, $5.50, payable in advance.)

The Utero-Ovarian Artery. Anatomy and Physiology with their Application in Diagnosis and Surgical Intervention. By Byron Robinson, M.D., Chicago. E. H. Colegrove, Chicago. 1903. (Price, $1.00.)

Medical and Surgical Report of the Presbyterian Hospital, New York City. Volume VI., January, 1904. Edited by Andrew J. McCosh, M.D., W. Gilman Thompson, M.D.

The Clinical Study of Blood-Pressure. A Guide to the Use of the Sphygmomanometer. By Theodore C. Janeway, M.D., Lecturer on Medical Diagnosis, University and Bellevue Hospital Medical College, New York. Small octavo, pp. 313. Illustrated. New York and London: D. Appleton & Company. 1904. (Price, $3.00.)

The Practical Medicine Series of Year Books. Ten volumes. Issued under the general editorial charge of Gustavus P. Head, M.D., Professor of Laryngology and Rhinology in the Chicago Post-Graduate Medical School. Volume VI. General Medicine. Edited by Frank Billings, M.D., and J. H. Salisbury, M.D., Chicago. Duodecimo, pp. 330. Chicago: The Year Book Publishers. 1903. (Price, $1.00; entire series, $5.50, payable in advance.)

Progressive Medicine. Volume VI., June, 1904. A Quarterly Digest of Advances, Discoveris and Improvements in the Medical and Surgical Sciences. Edited by Hobart Amory Hare, M.D., Professor of Therapeutics and Materia Medica in the Jefferson Medical College of Philadelphia. Philadelphia and New York: Lea Brothers & Company. (Per annum, in four cloth-bound volumes, $9.00; in paper binding, $6.00, carriage paid to any address.)

Materia Medica for Nursing. By Emily A. M. Stoney, Superintendent of the Training School for Nurses in the Carney Hospital, South Boston, Mass. Handsome duodecimo volume of 300 pages. Second edition, thoroughly revised. Philadelphia, New York, London: W. B. Saunders & Company. 1904. (Cloth, $1.50 net.)

Diseases of the Nose and Throat. By D. Braden Kyle, M.D., Professor of Laryngology and Rhinology, Jefferson Medical College, Philadelphia; Consulting Laryngologist, Rhinologist and Otologist, Saint Agnes's Hospital. Third edition, thoroughly revised and enlarged. Octavo volume of 669 pages, with 175 illustrations, and 6 chromo-lithographic plates. Philadelphia, New York, London: W. B. Saunders & Company. 1904. (Cloth, $4.00 net; sheep or half morocco, $5.00 net.)

### Index to New Series Volume XLIII.

(Whole Number Volume LIX.)

AUGUST, 1903–JULY, 1904.

PAGE.

ABSCESS, Retropharyngeal in Infancy ....................... 113
Acute Alcoholism ....................... 114
Adrenalin in Cardiac Toxemia of Pneumonia ........... 471
in General Surgery....... 408
Advertisements, Fraudulent and Filthy ....................... 628
Alaska, Coal and Oil Fields of....... 771
Albuminuria, Variations in Albumin. 467
Albumosuria ....................... 180
Amalgamation, Plan for............ 481
Anal Fissure, Cure of without Operation ....................... 117
Analgesia, Improvements in Method of Local ................... 189, 264
Anemia of Childhood.............. 112
Anesthesia, Local ................. 753
Antirheumatic Prescription ......... 609
Antitoxin Treatment, and Manufacture of ....................... 619
Antivivisection Libel Suit........... 486
Antonius, Notice of Appeal by....... 771
Apollinaris Water ................. 552
Appendectomy, Oxyuris Vermicularis in Appendix ..................... 178
Appendicitis, Conservative Treatment of .................. 392
Insurance against...... 202
Suppurating into Bladder ................ 470
Appendix, Absence of.............. 194
Army Service, Examinations for.... 467
Arsenic, an Improver of Nutrition... 749
in Tuberculosis............ 113
Use of ................... 748
Arteriosclerosis, Antisclerosin in.... 469
Association, New York State Medical, Final Action of.............. 629
Asthma, Pathology of and its Vicious Circle ............................ 381
Automobile, On the................. 743

PAGE.

BACTERIURIA ................. 115
Bagley, Thomas, Typhoid Fever. 657
Beahan, A. L., Uterine Myomata.... 581
Beta-Eucaine for Local Anesthesia.. 534
Betul-ol vs. Salicylate of Methyl.... 612
Bishop, Louis F., Prostration of the Circulation ....................... 670
Bladder Troubles of Old Men...... 297
Blind, Commission to Investigate Condition of Adult............... 698
Blood Pressure, Accurate Determination of .......................... 817
Books received........ 70, 143, 215, 285, 356, 430, 500, 568, 643, 715, 786, 844
Bright's Disease, Medical and Surgical Treatment of ............ 19
Ocular Manifestations in ........ 467
Brown, C. W. M., Easy Method of Percentage Infant Feeding........ 157
Brooklyn Medical Journal Invites Subscriptions .................... 772
Buffalo General Hospital, Reminiscences of ............... 538
Medical Journal, Complimentary to ............. 629

CALCULI in Kidney, Ureter and Bladder ...................... 307
Cancer, Increase in................. 684
Vital and Toxic Theories of. 14
Cardiac Hypertrophy ............... 825
Celiotomy, An Improper Term...... 754
Cheesman, W. S., Gastrojejunostomy for Pyloric Obstruction........... 244
Children, Neurasthenia in.......... 194
Cholelithiasis ...................... 601
and Cholecystitis, Diagnosis and Medical Treatment of........ 573

PAGE.
Circulation, Prostration of..........670
Clark, Clarence G., Treatment of Gonorrhea ..............442
Joseph C., Physician's Electrical Outfit and its Use...105
Clinton, Marshall, Perforation of Meckel's Diverticulum...730
Study of the Treatment of General Spreading Peritonitis ................823
Clowes, G. H. A., Vital and Toxic Theories of Cancer............. 14
Clubfoot, New Principle of Curing..399

**College and Hospital Notes:**

Albany Medical College.........839
Albany, Two New Hospitals at .........................136
Bradford (Pa.) Hospital.......419
Buffalo Children's Hospital.....559
Buffalo Fresh Air Mission Hospital .......................135
Buffalo General Hospital....... 14
Buffalo General Hospital Annual Report ..................778
Buffalo Homeopathic Hospital.. 64
Buffalo State Hospital Report...418
Canandaigua Physicians' and Surgeons' Hospital .............136
Emergency Hospital Medical Staff ......................572
German Deaconess's Hospital...560
Hagerstown (Md.) Hospital....560
D. W. Harrington Lectureship, University of Buffalo.........344
Hospital Appointments .........777
Lackawanna Steel Company Hospital .......................418
Massachusetts General Hospital ........................278
Medical College, University of Southern California ..........838
Medical Department, University of Buffalo ..............135, 278
Memphis Medical College Lectureship .....................209
New York School of Clinical Medicine ....................419
New York State Hospital for Crippled and Deformed Children ........................560
Riverside Accident Hospital....419
Riverside Hospital, Buffalo.....561
Thompson Memorial Hospital, Canandaigua ................838
Toronto and Trinity University Amalgamation ...............344
University Day, University of Buffalo .....................561

PAGE.
University of Buffalo Commencement .......................635

Congdon, Charles E., Personal Experience with Puerperal Fever....292
Consolidation, An Act to Authorise..483
Coroner, Passing of in New York...693
Coroner's Bill, New York City, Vetoed ............................771

**Correspondence:**

Afterthoughts—A. Jacobi.......756
Answers to Correspondents..... 44
Asthmatic Institute Regular—George N. Jack..............472
Letter from Berlin, Roswell Park.687
Medical Legislation at Albany—Frank Van Fleet............692
Optometry Bill once more—Frank Van Fleet ............472
Saint Louis Exhibit—Wm. R. Warner & Co. ................825

Curetment, Dangers of Uterine......261
Curtis, B. Farquhar, Surgical Treatment of Gallstones...............433
Cystitis ...........................512
in the Female..........263, 300
Cystoscopy, Fallacies of............464

DAGGETT, BYRON H., In Memoriam .....................461
Davis, William E. B., Monument to..698
Diarrhea, Enteroclysis in........... 40
Dignen, W. E., Cholelithiasis........601
Diphtheria, Antitoxin in............112
Dowd, J. Henry, Cystitis...........512
Dropsy, Its Therapeutics...........725
Dysemia, Clinical Notes on......... 30
Dysmenorrhea, Rheumatic..194, 732, 816
Treatment of .......614
"Dyspepsia," Surgical Treatment of..789

ECTOPIC Gestation, The Cervix in ......................752
Eddyism, Example of..............629
Effusions, Treatment of Serous.....750
Electrical Outfit, Physician's, and its Use ............................105
Empyroform, On...................191
Ethics, Change in Code of A. M. A..319
Examination, New York State Licensing, Successful Candidates, May and June, 1903..............118
Exodin, A New Purgative..........532
Explanation Regarding Change in Code of Ethics..................413

PAGE.

FAIRCHILD Bros. & Foster win Substitution Suit .............. 60
Formalin Disinfection, Methods of..535
Fractures of Leg, Treatment of by Massage and Passive Movements.. 82

GALLSTONES, Their Surgical Treatment ....................433
Glenny, W. H., Accurate Determination of Blood Pressure as an Aid to Diagnosis ....................817
Goelet, A. H., Palpation of Prolapsed Kidney and Method of Fixation ..............................233
Gonorrhea, Collargolum and Ung. Credé in ............611
Treatment of ..........442
Gonorrheic, A Youthful.............529
Gravel, Louis J., Clinical Notes on Dysemia ......................... 30
Griffith, Jefferson D., Fourteenth International Congress ............. 89
Gynecology, Specialty of, a "Goner".204

HAIG, ALEXANDER, Washing out Plan ....................185
Hanley, L. G., Appendectomy .......178
Hematocolpos, Congenital ............178
Myxosarcoma of Right Ovary ............177
Harrington, D. W., Reminiscences of Buffalo General Hospital..........588
Hayd, H. E., Fibroid Tumors of the Uterus ............145
Sterility and its Treatment ...............645
Heart Depression Following Antitoxin ............................112
Hedonal, Critical Clinical Study of..172
Heffron, John L., Our Relations to Problems in State Medicine.......217
Hematemesis Following Appendectomy ............................118
Hematocolpos, Congenital ..........178
Hemorrhoids, Treatment of.........114
Higgins, F. W., Conservative Treatment of Appendicitis .............392
Hip Disease, Diagnosis of...........400
Joint, Treatment of Acetabular Disease of ..................401
Method of Correcting Deformity of ..........................400
Hoffmann, Frank F., Dropsy, its Therapeutics .....................725
Howe, Lucien, Proposed Optometry Bill ..............................536
Hyaline Casts in Renal Diseases....468

PAGE.

**Illustrations:**

Barrett, William C., portrait...206
Clinton, Meckel's Diverticulum..731
Glenny, Sphygmomanometer.....818
Goelet, Prolapse of the Kidney:
Fig. 1, Palpation.............235
Fig. 2, " .............236
Fig. 3, " .............237
Fig. 4, The Goelet Suture....242
Hanley, Myxosarcoma of Right Ovary .........................178
Ingraham, Henry D., portrait...835
Jack, Vicious Circles of Asthma.383
LeBreton, Orthopedic Appliances:
Fig. 1. Corrective Machine for Flat-foot ...........306
Fig. 2, Leather Collar and Jacket ..............306
Fig. 3, Hip Spica ............306
Fig. 4, Long-leg Splint .......306
Fig. 5, Club-foot Shoes ......306
Fig. 6, Suspension Apparatus.306
Lewis, Squint and its Modern Treatment:
Fig. 1, Sketch of Neurons....249
Fig. 2, Worth's Amblyoscope..252
Lewis, Middle-ear Inflammation:
Fig. 1, Opening Tympanum...520
Fig. 2, Pus Filling Middle-ear.522
Fig. 3, Gauze Siphon in Position .................523
Fig. 4, Erosion into Mastoid..525
McMurtry, L. S., portrait.......827
Thompson, Justin G., portrait...632
Wey, Hamilton D., President Medical Society State of New York, portrait ...............547
Wyckoff, Cornelius C., portrait..339

INFANT Feeding, Easy Method of Percentage .....................157
Insane, Progress in care of........617
Iodine and its Compounds, Use of...733
Iritis .............................472

**Items:**

American Medical Association Prize Essay .................216
Antikamnia Chemical Co., calendar, 1904 ....................432
Battle & Company, Bacteriological and Blood Charts.........502
Breitenbach, M. J., Company, Yearbook, 1904 ..............432
Central Institute of Physical Therapeutics, Rome, Italy.....571
Daily Medical Journal, staff Correspondent Needed ..........360

PAGE.

Fairchild Brothers & Foster, Christmas Entertainment .....502
Fassett, Charles Wood, Medical Press Exhibit at World's Fair.502
Fellows Hypophosphite Co., Illustration of Therapeutic Conservatism ....................432
Mellier Drug Co., Medicine Man of the Sioux Indians.........572
New York Pharmacal Association, Facetiæ Medicorum......572
Oppenheimer Institute, Women's National Auxiliary ...........432
Palisade Manfg. Co., Actual Invalid in Tuberculosis ........432
Saunders, W. B. & Co., New Publications .................788
Schieffelin & Co., Flat Pocket Pencils .......................360
State Civil Service Examination.788
U. S. Civil Service Commission Examination .................432
Victor Koechl & Co., Acute Poisoning Chart ................502

JACK, Geo. N., Pathology of Asthma with Reference to its Vicious Circle ..........................381
Jackson, William H., In memoriam..458
Jewett, Charles Sherman, Medical and Surgical Treatment of Bright's Disease .......................... 19
Jones, William B., Bladder Troubles of Old Men .....................297

KERATOCONUS ..............372
Kidney, Movable ............755
Kidney, Palpation of Prolapsed and Method of Fixation of............233
Knee, Method of Correcting Flexion Deformity of....................400

LABORATORY for Study of Defective, Pauper and Criminal Classes ..........................554
Larynx, Bilateral Abductor Paralysis of ..............................225

**Leading Articles:**

Albany Medical Annals's Jubilee.484
American Medical Association at Atlantic City ................826
American Medical Association, Atlantic City, Lehigh Valley Railroad ......................696
Antivivisection Libel ...........410
Army Medical Reserve Corps...622

PAGE.

Buffalo Water Supply.......... 58
Calox, The New Dentifrice.....764
Commercialism in Charity......199
Dentifrices and the Teeth, Calox.694
Epidemic Impetigo Contagiosa..269
Faith Healers and the Law.....270
Fifty-fifth Annual Meeting, A. M. A.........................766
Grocer Druggists ...............769
Infectious Diseases and the Law.195
"Keep in Touch with Buffalo"..829
Lake Keuka Medical Association.128
Leo XIII.—Pius X.............129
Medical Ethics, Principles of....124
Medical License in Pennsylvania 52
Medical Organisations, Principles of .......................... 49
Medicine and Graft.............768
Neuropsychiatry, Recent Advances in ....................334
Ninety-eighth Annual Meeting, Medical Society State of New York .......................544
Ophthalmology versus Optometry .........................548
Paper Money and Disease......624
Pryor, Dinner to Dr...........626
Purification of the Mails........832
Roosa, Dinner to Dr............549
Shall the Society Publish a Journal? ........................551
State Society and Association not Rivals .......................126
Substitution, A Department Store Found Guilty of..............767
Substitution and Sudden Death.550
Unification Close at Hand......266
Unification Still Nearer.........480
University of Buffalo Commencement .........................761
Venereal Disease and the Canteen .........................625
Water Supply Again...........196

LeBreton, Prescott, Various Orthopedic Appliances .................304
Leucorrheal Discharges, Causation and Treatment of.................331
Leukocyte Count in Surgical Diagnosis ..............................364
Leukocytosis in Gynecology.........193
Levulose in Dietetics of Childhood..610
Lewis, Bransford, Calculous Formations in Kidney, Ureter and Bladder ..................307
G. Griffin, Keratoconus......372
F. Park, Modern Management of Middle-ear Inflammation.519
F. Park, Squint and its Modern Treatment ............248
Light Waves, Visible and Invisible.. 73

PAGE.

**Literary Notes:**

Arlington Chemical Co., Pathology of Typhoid Ulcer.........288
Arlington Chemical Co., The Law and the Doctor.......... 72
Bailliere, J. B. & Son, Paris, Scientific Bibliography ..........570
Battle & Co., Chart of Tumors..288
Breitenbach, M. J., Co., Bacteria Chart .......................144
Buffalo State Hospital, Thirty-second Annual Report........144
Cancer Laboratory, Fourth Annual Report .................571
Cincinnati Lancet-Clinic.........501
Daily Medical .................570
Daily Medical Journal..........359
Enno-Sander Prize .............360
Grafton Press, Ricketts's Surgery of Heart and Lungs.........431
International Journal of Surgery Co., Richards on Nose and Throat ....................144
International Medical Magazine.570
Journal of Infectious Diseases... 72
Journal of Tuberculosis.........359
Los Angeles Medical Journal....431
Lydston, G. Frank, Diseases of Society .....................216
Mariani's Coca Leaf...........569
Maryland Medical Journal..571, 572
Medical Book News............571
Medical Fortnightly............501
Medical Library and Historical Journal .....................569
Medical Mirror ................144
Medical Recorder...............502
Polk's Medical Register and Directory ......................846
Practical Medicine, Delhi, India.431
Treat, E. B., & Co., Announcement .......................360

Loveland, B. C., Relation of Fat to Nervous Diseases ................289

MAMMARY Inactivity, Thyroids in .........................260
Mann, Matthew D., Early Days of Ovariotomy .....................503
McCormack, J. N., Plea for Unification and Uniform Organisation...474
McMurtry, L. S., Perfected Treatment of Fibroid Tumors of the Uterus ..........................402
Medical Congress, Fourteenth International .................. 89
Practice, Curiosities of...... 1
Research, Apathy Regarding.412

PAGE.

Medicine, Unlawful Practice of.....753
Medicines and Foods, National Bureau of .........................413
Meckel's Diverticulum, Perforation of ..............................730
Mickle, Herbert, In Memoriam......460
Middle-ear Inflammation, Management of ........................519
Milk Glands, Long Continued Function of .........................259
Millener, F. H., Light Waves, Visible and Invisible .................... 73

**Miscellany:**

Blakiston's Son & Co., Deaver's Surgical Anatomy ...........716
Davis, Nellie S. Directory of Nurses ..................... 72
Dios Chemical Co., Visiting List.716
Maltine Co., Visiting Lists......716
Palisade Manufacturing Co., Declaration of Independence......716
State Civil Service Examinations.644

Mynter, Herman, In Memoriam.....452

NEGRO Population Paradox.....204
Nervous Diseases, Relation of fat to ..........................289
Neuralgia .........................748
Newspaper Medicine Paragraphs....337
Niagara Falls, Report of Health Officer of ........................693
Nurses, State Board of Examiners of Registered ...............203
State Registration of....... 60

**Obituary:**

Andrews, Lewis B..............837
Andrews, Myron H.............836
Baker, Washington H..........703
Bair, E. A.....................775
Barrett, William C.............206
Bartholow, Roberts ............775
Birmingham, J. J...............341
Bissell, Wilson Shannon........275
Blair, Frank P.................775
Brackett, Martin E.............557
Broadt, Peter ..................490
Cosford, Thomas B............634
Cowles, Andrew L.............634
Cushing, Clinton ...............775
Daggett, Byron H..............489
Davis, Nathan Smith...........846
Duff, John Milton..............775
Edson, Cyrus ...................416
Engelmann, George J...........341

PAGE.
Gaston, James McFadden.......340
Goltry, George Humphrey.......776
Gordon, Pliny P................634
Haney, Matthew F ..............634
Hardon, Virgil O...............633
Hibbard, James F...............207
Higgins, Francis W.............416
Ingraham, Henry Downer..775, 835
Jackson, Frank S...............490
Jackson, William H.............416
Jones, George G................557
Kibbe, Alfred B................702
LeVan, Clarence B..............131
Loop, Dennis D.................703
Lundy, John B..................208
McCray, Guilford W.............702
McCorn, William A..............634
McDermott, William J...........634
McGarry, James ................131
Maclean, Donald ...............132
Mercer, William M..............131
Mickle, Herbert ............... 62
Miller, DeLaskie .............. 62
Milring, Edwin A...............416
Murray, Charles A..............275
Newman, Robert ................275
Nutten, Wilbur S...............702
Paine, Horace M................415
Parker, Sylvanus E.............702
Patchin, Robert A..............208
Perine, Francis Marion......... 62
Playfair, William S............131
Polk, J. Metcalf...............702
Richardson, Alonzo B........... 63
Richmond, Charles H............634
Rochester, Thomas M............ 63
Smith, Austin D................702
Southard, William B............634
Taylor, Horace C...............416
Thompson, Sir Henry............702
Thompson, Justin G.............632
Thornton, J. Knowsley..........556
Waid, A. P.....................417
Walker, Delavan E..............837
Warren, S. H...................341
Warren, William M..............417
West, Hamilton A...............490
Wilding, Robert G..............557
Wyckoff, Cornelius C...........339

OPERATIONS, Justifiableness of Exploratory .................595
Optometry Bill, Hearing on.........697
Bill Proposed ...........536
Bills, Protest Against....486
Orthopedic Appliances ..............304
Osteopathic "Ethics" ...............203
Osteopathy, Status of...............616
Ovariotomy, Early Days of...........503
Ovary, Myxosarcoma of...............177

PAGE.
PANAMA Canal, Sanitation of...628
Park, Roswell, Letter from on German Congress of Surgeons....699
Park, Roswell, Surgical Treatment of "Dyspepsia" .....................789
Pennyroyal, Poisoning from........193
Peritonitis, Treatment of General Spreading ......................823

**Personal.**

Auel, C. H. W.; Roe, John O.; Meyer, Edward, J.; Hanley, L. G.; Bull, William T.; McMurtry, L. S.; Foshay, P. Maxwell ........................701
Brewer, George E.; Evans, William A. ......................699
Brown, John Young; Briggs, Albert H.; Johnston, George Ben; Glosser, H. H.; Pantzer, Hugo O.; Ross, James F. W. 61
Brush, Edward F.; Beach, Eugene; Westmoreland, W. F.; Westinghouse, Geo. H.; Meade, Charles H. B.; Park, Roswell; Krauss, Wm. C..............414
Bush, Robert P.; Whitney, Lee A.; Park, Roswell; Koehler, Edward E.; Halbert, John S.; McGuire, F. W.; Trick, Harry R.; Babcock, Warren L.; McGuire, E. R..................774
Cary, Charles; Callahan, Wm. H. ........................ 62
Crego, Floyd S.; House, William; Krauss, William C.; Gregory, Willis G.; Vaux, Charles L. ..................487
Darlington, Thomas; Woodbury, John McGaw; Smith, Chauncey Pelton; Ross, James F. W.; Swift, E. G.; Reed, Charles A. L.; Ingraham, Henry D.......488
Dorsett, Walter B.............204
Draper, Andrew S.; Park, Roswell; Gaylord, Harvey R.; Putnam, James W.; Cutting, Reger; Wheeler, David; Peterson, Frederick ............631
Haase, Charles; Johnson, Ray H.; Millener, F. H.; Matzinger, H. G.; Smith, T. Guilford; Mann, Arthur E..............415
Johnston, George Ben; Wende, Ernest; Lewis, F. Park; Barrett, Frederick J.; Peckham, Cyrus P.; Bonifield, Charles L.; Dudley, E. C.; Howe, Lucien .....................834

PAGE.

Mann, Edward C.; Gray, John R.; Edmunds, H. M. . . . . . . . . . . 338
Mann, Matthew D.; Mathews, Joseph M.; Reed, Charles A. L.; McMurtry, L. S. . . . . . . . . . . 131
McCormack, J. N.; Wilson, Nelson W.; Miller, Henry N. . . . . . . 555
Mikulicz, Prof. J.; Smith, Eugene A. . . . . . . . . . . . . . . . . . . . . 489
Millener, F. H.; Wilcox, Dewitt G.; Pryor, J. H.; Pryor, William R. . . . . . . . . . . . . . . . . . . . . . 556
Park, Roswell; Brownell, H. B.; Banta, Charles W.; Hinkel, F. Whitehill; Buswell, H. C.; Grant, Henry Y.; Pohl, Gustav A. . . . . . . . . . . . . . . . . . . . . . 700
Park, Roswell; Ferguson, A. H.; McGuire, F. W.; Snow, Irving M.; Randall, Lillian Craig; Preiss, Frederick . . . . . . . . . . . . 130
Ramsey, Maitland . . . . . . . . . . . . . . 846
Reed, Charles A. L.; Brush, Edward N.; Smith, Chauncey Pelton . . . . . . . . . . . . . . . . . . . . . . 770
Shurly, E. L. . . . . . . . . . . . . . . . . . . . 339
Smith, Chauncey Pelton; Thornton, Wm. H.; Green, Walter D.; Taber, P. E.; Hendee, Lawrence; MacDonald, Carlos F. . . . . . . . . . . . . . . . . . . . . . . . . . . 274
Smith, Chauncey Pelton; Clowes, G. H. A. . . . . . . . . . . . . . . . . . . . . 572
Smith, Eugene A.; Crockett, M. A.; White, Wm. A.; Benedict, A. L.; Bainbridge, W. S.; Woodruff, J. J.; Snow, Irving M. . . . . . . . . . . . . . . . . . . . . . . . . . . 205
Smith, T. Guilford; Vander Veer, A.; Wood, Leonard . . . . . . . . . 630
Stockton, Charles G.; Park, Roswell; Hoffa, Albert . . . . . . . . . . . 833
Thornton, William H.; Thornton, William Irving; Knisley, A. B.; Twohey, John J.; Wheeler, David; MacDonald, J., Jr.; Merriman, W. E.; Spratling, William P.; Haskell, Cameron; Mabon, Wm. . 773

Pleuritic Effusion, New Sign of, in Children . . . . . . . . . . . . . . . . . . . . . . . . . 111
Pneumonia, Cardiac Tonics in. . . . . . 746
  Prevalence of . . . . . . . . . 697
Post Check Currency and Clean Money . . . . . . . . . . . . . . . . . . . . . . . . . 554
Potter, William Warren, Diseases of Respiratory Tract from Pension Examiner's Viewpoint . . . . . . . . . . 163

PAGE.

Professional Life, The Scholar in. . . 717
Professorship in Medical History. . . . 273

**Progress in Medical Science.**

Genitourinary and Syphilitic Diseases, by Byron H. Daggett, 115, 180, 262, 462
Genitourinary and Syphilitic Diseases, by Nelson W. Wilson. . 529
Obstetrics and Gynecology, by Maud J. Frye. . . . . . . . . . . . . . . . 259
Orthopedics, by Prescott LeBreton . . . . . . . . . . . . . . . . . . . . . . . . . 399
Pediatrics, by Maud J. Frye. . . . . 111
Therapeutic Notes, by Nelson W. Wilson . . . . . . . . . . . . . 113, 746

Prostatectomy, Indications for. . . . . . 466
  Suprapubic . . . . . . . . . 531
  Technic of . . . . . . . . . 462
Prostate, Surgery of. . . . . . . . . . . . . . . 262
Prostatic Hypertrophy . . . . . . . . . . . . . 465
Public Health and Marine Hospital Service, Examinations for. . . . . . . . 542
Puerperal Fever, Personal Experience with . . . . . . . . . . . . 292
  Sepsis, Iodine Treatment of . . . . . . . . . . . . . . . . . . . . 261
  Sepsis, Treatment of. . . . . 328
Pyloric Obstruction, Gastrojejunostomy for . . . . . . . . . . . . . . . . . . . . . . . 244

REED, Charles A. L., and the Code of Ethics . . . . . . . . . . . . . . 337
Reed, Walter, Memorial Fund. . . . . . 200
Regents, New Board of, Organised. . 770
Respiratory Tract, Some Diseases of. 163
Rheumatism, Acute . . . . . . . . . . . . . . . . 531
  Chronic . . . . . . . . . 333, 750
  Prescription for. . . . . . . 448
Ricketts, B. Merrill, Typhoid Gangrene of the Lower Extremities. . 361

**Reviews.**

Abrams: The Blues . . . . . . . . . . . . 783
Anders: Practice of Medicine. . 497
Archinard: Microscopy and Bacteriology . . . . . . . . . . . . . . . . . . . . 355
Ashby: Diseases of Women. . . . 346
Babcock: Heart and Arterial System . . . . . . . . . . . . . . . . . . . . . 69
Babcock-Somers: Preventive Medicine, Two Prize Essays. . 780
Bandler: Urine and Tubal Gestation . . . . . . . . . . . . . . . . . . . . . . 69
Barton: Thesaurus of Words and Phrases . . . . . . . . . . . . . . . . 351

PAGE.
Baruch: Hydrotherapy ........842
Bell: Worth of Words.........839
v. Bergmann: System of Practical Surgery, Vol. I...........840
Bickham: Operative Surgery....419
Biggs: Bovinine ..............714
Blakiston: Physicians' Visiting List for 1904................429
Bridge: Tuberculosis ..........211
Buck: Principles of Otology...424
Buck: Reference Handbook of Medical Sciences, Vol. VI., 210; Vol. VII. ..............639
Button: Mother and Daughter..779
Cabot: Physical Diagnosis......424
Casper-Richter: Diagnosis of Kidney Disease .............426
Chapman: Medical Jurisprudence, etc. .................... 69
Cheyne-Burghard: Manual of Surgical Treatment Vol. VII. ........................136
Church-Peterson: Nervous and Mental Diseases ............563
Cohen: Physiologic Therapeutics, Vol. X., 67; Vol. VIII., 282; Vol. VII. ..............710
Crandall: How to Keep Well... 68
Crile: Blood Pressure in Surgery ........................635
DaCosta: Modern Surgery......565
Davis: Self-Cure of Consumption ........................783
Dawbarn: Treatment of Malignant Growths ................707
Dorland: Pocket Medical Dictionary ......................430
Dorland: Medical Dictionary....709
Douglas: Surgical Diseases of the Abdomen ................278
Dunglison: Dictionary of Medical Science ................425
Dwight: Medical Jurisprudence.354
Edgar: Practice of Obstetrics...421
Egbert: Hygiene and Sanitation ........................707
Eisendrath: Clinical Anatomy..638
Ellis: Sexual Impulse in Women ........................350
Engelhard: Standard Medical Directory ...................426
Ewing: Pathology of the Blood.640
Findlay: Diseases of Women...210
Flick: Consumption ...........353
Fox: Diseases of the Eye......705
French: Practice of Medicine...566
Galbraith: Four Epochs of Woman's Life ..................429
Gilliam: Textbook of Gynecology ........................561

PAGE.
Gould: Yearbook of Medicine and Surgery, 1904 ...........638
Gould: Biographic Clinics, Vol. II. ..........................779
Gowers: Lectures on Nervous Diseases ...................641
Griffith: Care of the Baby...... 68
Haab: Diseases of the Eye......710
Hale: Anatomy ...............708
Hammer: Radium and Radio—Active Substances ...........352
Hare: Progressive Medicine, Fifth Series, Vol. II., 67; Vol. III., 353; Vol. IV., 498; Sixth Series, Vol. I................784
Head: Practical Medicine Yearbooks, Second Series, Vol. V., Obstetrics, 213; Vols. VI. and VII., Medicine and Pediatrics, 345; Vol. IX., Physiology, etc., 352; Vol. VIII., Materia Medica., etc., 355; Vol. X., Skin and Venereal Diseases, 499; Vol. III., Eye, Ear, Nose and Throat, 782; Third Series, Vol. II., General Surgery .........843
Hill; Textbook of Chemistry...347
Hirst: Diseases of Women......495
Hirst: Textbook of Obstetrics..564
Howe: Expectant Mother ...... 70
Howe: Handbook of Parliamentary Usage ...................778
Hughes: Neurology and Psychiatry .........................712
Juler: Ophthalmic Science and Practice .....................782
Keen-White: American Textbook of Surgery .............349
King: Manual of Obstetrics....352
King: Medical Union, No. 6....843
Knight: Diseases of Nose and Throat .....................427
Kyle: Diseases of Nose, Throat and Ear ...................784
Lea: Medical News Visiting List for 1904 ..... ..... .........353
Lippincott: International Clinics, Thirteenth Series, Vol. I., 66; Vol. II., 284; Vol. III., 428; Vol. IV. ....................642
McFarland: Pathogenic Bacteria ........................428
McGlannan: Organic and Physiologic Chemistry .............708
McGlannan: Physics and Inorganic Chemistry .............567
Martin: Manual of General Pathology .....................841
May: Diseases of the Eye......499

PAGE.
Medical Directory of New York, New Jersey and Connecticut. .354
Morrow: Social Diseases and Marriage ....................706
Mosher: Testing Nerves and Muscles ......................642
Nobles: Minor Surgery and Bandaging ....................212
Nothnagel: Encyclopedia of Medicine, Vols. V. and VI... 64
Ogden: Examination of Urine. .497
Opie: Disease of the Pancreas. .283
Overall: Diseases of the Prostate ........................495
Palmer: Surgical Asepsis......284
Peterson-Haines: Legal Medicine and Toxicology, Vol. I. . .139
Potter: Compend of Human Anatomy ....................355
Pryor: Gynecology ............281
Pusey-Caldwell: Röntgen Rays in Therapeutics and Diagnosis.140

Reports:

Commissioner of Education, Vol. II., 1900-01, 66; Vols. I. and II., 1902...........713
Johns Hopkins Hospital Report, Vol. XI., 1903.......500
Michigan State Board of Health, 1901 .............143
Mt. Sinai Hospital Report, Vol. III., 1901-02.........712
N. Y. State Department of Health, 1901 ............214
Public Health and Marine Hospital Service, 1902....841

Richards: Nose and Throat....498
Robinson: Latin Grammer of Medicine and Pharmacy......351
Rockwell: Medical and Surgical Uses of Electricity...........141
Roger: Infectious Diseases.....423
Schaeffer: Operative Gynecology.709
Schamberg: Compend of Skin Diseases ....................427
Scudder: Treatment of Fractures ........................781
Smith: Nurses' Guide to Bandaging and Dressing ..........356
Snow: Uses of Röntgen Ray...422
Starr: Organic Nervous Diseases ........................138
Stedman: Twentieth Century Practice, Vol. XXI........... 65
Stengel: Textbook of Pathology.496
Stevens: Practice of Medicine. .567

PAGE.
Stevens: Materia Medica and Therapeutics ................212
Stoney: Practical Points in Nursing ..................... 70
Suter: Refraction and Motility. 68
Taylor: Pocket Account Book. .356
Thayer: Compend of Pathology.784

Transactions:

American Association of Obstetricians and Gynecologists, Vol. XV., 1902......214
American Dermatological Association, 1902, 142; 1903, 778
American Gynecological Society, 1903 ..............785
American Laryngological Association, 1903 ...........498
American Otological Society, 1903 ......................714
American Röntgen Ray Society, 1902 ..............142
American Surgical Association, Vol. XXI., 1903.....715
Medical Association of Alabama, 1903 ..............713
Medical Society State of New York, 1903..........348
National Association of U. S. Pension Examining Surgeons, 1902-03............565
N. Y. Academy of Medicine, 1896-1901 ................785
Southern Surgical and Gynecological Association, Vol. XV., 1902 ................285
Texas State Medical Association ...................786

Treat: International Medical Annual, 1904 ...................711
Veasey: Diseases of the Eye... 67
Von Noorden: Metabolism and Nutrition, Part IV...........643
Warren: Principles of Obstetrics .........................350
Wathen: Normal Histology ....711
Webster: Textbook of Obstetrics .........................493
Wells: Compend of Gynecology.712
Wheeler: Hints for Busy Mothers ..........................356
Whitman: Orthopedic Surgery.494
Williams: Manual of Bacteriology ........................641
Wilson: Medical Pocket Formulary ........................780
Witthaus: Urinalysis and Toxicology ......................354

PAGE.

Wood: Diseases of the Eye......781
Wood: Medical Record Visiting List for 1904................429
Worth: Squint ................213
Zapffe: Manual of Bacteriology.212
Ziegler: General Pathology ....640

SCHULTZE, B. S., Problem of Determination of Sex..........183
Sciatica ..........................749
Serpents, Bites of.................770
Sex, on Determination of..........183
Signed Editorials .................274
Smallpox, A Menace................485
Smith, Chauncey Pelton, Curiosities of Medical Practice........ 1
 Edward T., Animal Therapy in Tuberculosis ...........650
 Eugene A., Justifiableness of Exploratory Operations.595
 Frank Hyatt, Scholar in Professional Life ............717

**Society Meetings:**

American Academy of Medicine.704
American Association of Obstetricians and Gynecologists, 132, 276, 776, 837
American Congress on Tuberculosis ....................... 134
American Electro-Therapeutic Association .............135; 491
American Institute of Homeopathy .......................838
American Medical Association and other Atlantic City Meetings .........................776
American Medico-Psychological Association .............492, 557
American Microscopical Society.777
American Public Health Association ....................208, 493
Buffalo Academy of Medicine, 209, 277, 342, 417, 492, 559, 703, 776
Buffalo Academy of Medicine, Laryngo-Otological Section....635
Buffalo Physicians' Club........635
Central New York Medical Association .............63, 135, 209
Erie County Medical Association, 278, 418
French Congress of Climatotherapy and Hygiene .......491, 558
Homeopathic Medical Society of Western New York..........704
Lake Erie Medical Society, 63, 277, 704
Lake Keuka Medical and Surgical Association .............. 63

PAGE.

Medical Society County of Chautauqua .......................341
Medical Society County of Chemung .......................704
Medical Society County of Erie, 418, 777
Medical Society of Missouri Valley ..........................558
Medical Society State of New York ...............135, 209, 341
Mississippi Valley Medical Association ...........134, 276, 491
N. Y. State Association of Railway Surgeons ..............345
National Association of Pension Examining Surgeons .........558
Roswell Park Medical Club.....276
Seventh International Congress of Otology ..................491
Southern Surgical and Gynecological Association..135, 342, 491
Tenth Quadrennial Congress of Polish Physicians........558, 635
Vanderburgh County (Ind.) Medical Society .............558
Western Surgical and Gynecological Association ......345, 493

**Society Proceedings:**

Alumni Association, University of Buffalo ...................736
Buffalo Academy of Medicine, 36, 179, 254
 Section on Medicine, 253, 317, 398, 527, 678, 680
Central New York Medical Association .....................312
Lake Keuka Medical and Surgical Association ..............109
Medical Society County of Erie, 34, 255, 315, 449, 681
Medical Society of the State of New York ..................256
N. Y. Odontological Society....682
N. Y. State Medical Association, Special Meeting .............672
Roswell Park Medical Club.....733

Spine, Operation for Fracture of..265
Squint and its Modern Treatment..248
Standards, Bureau of...............554
State Medicine, Problems in........217
Sterility and its Treatment..........645
Stockton, Charles G., Diagnosis and Medical Treatment of Cholelithiasis and Cholecystitis .............573
Sublamine, A Conjunctival Disinfectant .........................407
Syphilis, Mercury in Treatment of..116

PAGE.
TETANUS and the Toy Pistol..272
Tonsillitis ....................265
Töpfer, A., Critical, Clinical Study of Hedonal ....................172
Toy Pistol Evil....................201
Trikresol in Paraurethral Abscess.. 42
Tuberculosis, Animal Therapy in...650
Congress ............485
Typhoid Fever ................657, 747
Fever Epidemic at Butler, Pa. ..................412
Fever Epidemic at Watertown, N. Y............553
Fever, Unguentum Credé in.755
Gangrene of the Lower Extremities ............361
Perforation ...............740

ULCERS, Treatment of Indolent..752
Unification, Plea for..........474
Unification Ratified ................699
Urban, A. H., Bilateral Abductor Paralysis of the Larynx .........225
Urotropin, on.....................685
Versus Helmitol .......754
Uteri, Treatment of Carcinoma.....751
Uterine Myomata ..................581
Uterus, Bacteriology of the Puerperal ....................261
Fibroid Tumors of.........145

PAGE.
Perfected Treatment of Fibroid Tumors of.........402

VACCINATION, Present Status of ..........................809
Valentine Chemical Co., Reprimanded by Dr. F. C. Valentine.........555
Van Duyn, E. S., Influence of Leukocyte Count in Deciding for or against Operation ...............364
Varicocele ........................466
Velpeau, Anecdote of...............771
Vertebral Arches, Primary Tuberculosis of ........................401
Vital Statistics, Improvement in....336

WALLACE, W. L., Cystitis in the Female .................300
Washing out Plan (in Uric Acid Excess) ..........................185
Wey, Hamilton D., Present Status of Vaccination .....................809
Wheeler, David E., Treatment of Fractures by Massage and Passive Movements ...................... 82
Wyckoff, C. C., In Memoriam......315

YELLOW Fever at Tampico.....204

